# Quality of life and clinical-radiological long-term results after implant-associated infections in patients with ankle fracture: a retrospective matched-pair study

**DOI:** 10.1186/s13018-017-0608-x

**Published:** 2017-07-17

**Authors:** Patrick Ziegler, Donat Schlemer, Ingo Flesch, Sonja Bahrs, Ulrich Stoeckle, Sebastian Werner, Christian Bahrs

**Affiliations:** 1 Department of Traumatology and Reconstructive Surgery, BG Trauma Center Tübingen, University of Tübingen, Schnarrenbergstr 95, 72076 Tuebingen, Germany; 2 Department of Diagnostic and Interventional Radiology, University of Tübingen, Hoppe-Seyler-Str. 3, D-72076 Tübingen, Germany; 3 Department of Orthopedics, Traumatology and Sports Medicine, Bogenhausen, Kliniken München GmbH, Englschalkinger Str. 77, 81925 Munich, Germany

**Keywords:** Ankle, Infection, Osteosynthesis, Outcome

## Abstract

**Background:**

Ankle fractures are frequently occurring injuries. Despite the relatively simple operative technique, patients often suffer from postoperative complications. Little is known about postoperative treatment of implant-associated infections of the ankle. Therefore, this study shows and evaluates a treatment algorithm in long- and short-term outcomes compared to infection-free patients.

**Methods:**

Data from patients of over 20 years of a level 1 trauma center and university hospital was retrospectively analyzed including age, gender, comorbidities, smoking status, fracture classification, number of revisions, length of in-patient stay due to fracture and infection, and results of microbiological specimen with the length of antibiotic treatment. Moreover, present long-term outcome was evaluated by the American Orthopaedic Foot and Ankle Society (AOFAS) hindfoot score, the Ankle Osteoarthritis Score, and the Short Form 36 score and compared to a matched-pair infection-free patient cohort.

**Results:**

Forty-four patients could be retrospectively evaluated (51% male, 49% women, mean age 46 ± 17 years). Most of the cases were Weber B fractures (38%) following an in-patient stay from 51 ± 4.3 days after primary treatment and 77 ± 10.0 days after secondary treatment in our hospital. Microbiological specimen showed in 77% *Staphylococcus aureus* with following intravenous antibiotic treatment for 13.9 ± 3.1 days in mean. Common comorbidities/risk factors were cardiovascular disease (28%), smoking (15%), and diabetes (18%). Cure of infection and clinical and radiographic osseous consolidation could be documented for all cases.

Patients with implant-associated infections had significantly more risk factors than infection-free patients (1.1/0.33; *p* = .02 per patient). The matched-pair group showed significantly better long-term outcome in mean regarding the Ankle Osteoarthritis Score (2.0 ± 1.2/13.9 ± 4.7) and AOFAS hindfoot score (96.7 ± 1.9/87.3 ± 3.4).

**Conclusion:**

Immediate revision surgery with aggressive debridement, microbiological diagnostics, antibiotic therapy, and use of a drain until osseous consolidation is reached with following removal of the implant in patients with implant-associated infections after ankle fracture and open reduction internal fixation lead to cure of infection and fair long-term outcome in all cases. Special care must be taken of risk factors like diabetes and smoking.

**Trial registration:**

24/2008BO2

## Background

Among fractures treated by trauma surgeons, ankle fractures are the common injuries with an incidence of 187:100,000 people in the US population. However, more frequently, they occur in patients with comorbidities such as diabetes mellitus, peripheral arterial disease, and osteoporosis [1–3].

For surgical treatment of displaced ankle fractures, various implants are commonly used in daily practice. Plates, screws, Kirschner wires, and also external fixations are represented [4, 5]. Still, surgery is performed in most of the cases with open reduction and plate osteosynthesis. Despite the relatively simple surgical technique, this intervention is often associated with complications. Documented complications in the literature are postoperative wound healing complications, infections, postoperative malalignment, posttraumatic arthritis in the long-term follow-up, and (septic) non-union [6, 7]. Especially postoperative infections are reported in the literature with a frequency between 1 and 8% [8–11]. Patients with diabetes mellitus suffer from up to three times more postoperative complications than non-diabetic patients [10, 12–14]. Implant-associated postoperative infections are a rare complication after surgical treatment of ankle fractures in the past. Caused by the increasing number of fractures and multi-morbid patients, currently, infection numbers absolutely increase [15, 16]. The development of an implant-associated infection can be a potentially devastating complication following foot and ankle surgery. This leads to a high number of revision surgeries after initial fracture stabilization and potential loss of function of the joint and quality of life in the long-term follow-up [9]. The aims, however, of treating implant-associated infections are controlling of the acute infection and osseous consolidation and prevention of chronic osteomyelitis [17]. Regarding the management of an implant-related infection after ankle fracture, several methods are mentioned in the literature. These are conservative treatment with local or systemic antibiotics, operative revision and change of the implant, or revision and leaving a drain in place until osseous consolidation [18–20].

Reviewing the literature according to implant-related infection of the ankle joint after fracture stabilization demonstrated neither an adequate consensus nor any data on long-term clinical and radiological results after treatment [21]. Therefore, the aim of the present study was to evaluate a calculated treatment of patients with implant-associated infections after displaced ankle fracture. A long-term standardized treatment in cases of implant-associated infections with revision surgery and insertion of a drain until osseous consolidation was performed in our clinic. Target parameters were the number of revision surgeries, the length of in-patient stay, the amount of stated risk factors during the course, and the functional, radiological, and quality of life-based long-term score results using the Ankle Osteoarthritis Score, the American Orthopaedic Foot and Ankle Society (AOFAS) hindfoot score, and the Short Form 36 (SF-36) score [22, 23].

## Methods

The study is a retrospective longitudinal clinical-radiological analysis based on paper and electronic data from a clinic’s registry. The study was conducted in a clinic for trauma surgery serving as a trans-regional level 1 trauma center and university hospital. It includes a specialized department for septic surgery with a high rate of patient transfers from other hospitals. The clinic’s documentation system was analyzed regarding all patients who underwent surgical treatment because of implant-associated infections after ankle fracture in a period of 20 years.

Besides the epidemiological data such as gender, age, comorbidities, smoking status, fracture classification, the number of revisions, and the length of in-patient stay due to fracture and infection management, we documented the results of microbiological swab with the length of antibiotic treatment. An implant-associated infection was determined with clinical infection signs such as increased pain, swelling, redness or warmth around the affected area, inflammatory laboratory markers (e.g., CRP and leukocytes), and a positive wound or intraoperative microbiological swab. Reviewed comorbidities were diabetes, cardiovascular diseases, thyroid disease, dermatological diseases, neurological diseases, rheumatoid diseases, immune suppression, and other metabolic diseases. The outcome was evaluated with the Ankle Osteoarthritis Scale and the AOFAS hindfoot score [24, 25]. For each group, infection patients and matched-pair patients, the SF-36 questionnaire was evaluated, too. The 8-scale profile of the SF-36 includes vitality, physical functioning, body pain, general health perceptions, physical role functioning, emotional role functioning, social role functioning, and mental health [22].

Exclusion criteria were patients with superficial wound infections (fistula of suture materials, necrosis of the edge of the wound), open fractures, and fractures which were not initially treated with a plate osteosynthesis.

### Study design

The whole cohort of detected infection patients from the registry was evaluated due to above-mentioned risk factors and patient’s history. Both patients who underwent the whole treatment (from initial fracture stabilization followed by revision(s) due to implant-associated infection and persistent drain with implant removal) in our hospital and patients who were transferred to our clinic, with an implant-associated infection after initial ORIF of an ankle fracture, for further surgical treatment including persistent drain in our institution were involved in this cohort.

After evaluation of these patients, an infection-free matched-pair group was built to compare the characteristics and the outcomes to patients without any complications after ankle fracture and ORIF. The criteria for the matched-pair patients were:

- Identical classified fracture of the ankle due to AO Foundation fracture classification (classified by a specialist for radiology and a specialist for trauma surgery)
- Age should not differ by more than ±5 years from the patient’s age with plate-related infection
- Identical gender
- Time of injury should not diverge by more than ±3 years
- Same method of initial operation
- Similar comorbidity status regarding cardiovascular disease, diabetes, smoking, and obesity

### Management of implant-associated infections after surgical stabilization of ankle fractures

**Fig. 1:**
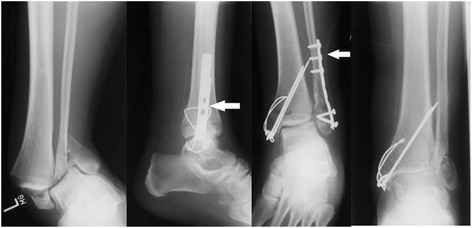
X-rays of an ankle fracture before and after ORIF with a drain left in place until osseous consolidation and following implant removal

In our clinic, a standardized surgical management of implant-associated infections after ankle fractures was performed. Therefore, the infected wound was revised and diagnostic biopsy for microbiological evaluation was taken, followed by aggressive debridement and removal of pus, necrotic tissue, dead bone, and abscess membranes. The implant from the initial stabilization procedure was left in place in all cases as osseous consolidation was not reached yet. If the soft tissue findings allowed a tension-free closure of the wound, a 10- or 12-CH drain with perforations along the whole length of the implant was inserted and the wound was closed. In cases with soft tissue inflammation, swelling, and, therefore, high tension, a sequential vacuum sealing was performed. In these cases, patients had to undergo revision surgery in normally 5–7 days with initiation of the drain therapy in dependence of the soft tissue inflammation and tension. All patients were immobilized with a splint or a cast with 20 kg weight-bearing for the time of fracture healing and therapy. The drain was mobilized every day to prevent clotting due to debris and loss of suction of the drain either by an out-patient care service or by the patients themselves. In this time, no drain was accidentally lost. At the beginning, all patients got either cefazolin or, if they showed any allergies, clindamycin as calculated antibiotic therapy. As the diagnostic biopsy results were available, the antibiotic therapy was changed according to the antibiogram. Then, with sufficient osseous consolidation after normally 10–12 weeks, further treatment has to be determined. Standardly, an X-ray examination in two planes was used for determination of the osseous consolidation. The fracture was considered as consolidated if at least 75% of the fracture gap was filled with bone and the patient had no pain around the gap in the clinical examination. Normally, the drain was removed at that time. If the patient showed up again with a local re-infection, a revision with radical debridement and removal of the implant was performed. If there was no re-infection after removal of the drain with osseous consolidation, the therapy was finished. A re-evaluation was planned with a possible implant removal if the patient reported any implant-related problems at a subsequent date (Fig. 1).

### Statistics

To ensure a high level of structural equality, a matched-pair technique was used to build study groups. The tests for matched-pair analysis were performed with the McNemar test for dependent samples. To avoid randomly occurring significances, the Bonferroni-Holm correction for multiple testing was performed. To compare the score outcome in both groups, the *t* test was used. The significance level was respectively reported at *p* < .05.

## Results

For this retrospective analysis, the data of 44 patients were reviewed. Five patients (11%) died because of other diseases and were not included for further evaluation. Twenty-one of 39 (54%) investigated patients were treated initially at our hospital. Eighteen (46%) patients had an initial at an external institution, following further operative treatment in our hospital. These patients were referred to our hospital because it includes a specialized department for septic surgery.

Twenty-one patients could be examined again at our clinic with a complete follow-up to evaluate the long-term outcome and compare the results to a matched-pair group. The significances in criteria for the matched-pair group were between *p* = .95 and *p* = 1.0. The general gender distribution was 20 (51%) men and 19 (49%) women with a mean age of 46 ± 17 years.

The injury pattern leading to ankle fracture was in 34 (82%) patients at a low-energy trauma. A high-energy trauma was documented in five (12%) patients.

The injury pattern leading to operative treatment of the ankle was in 38% of the patients, a Weber B fracture, and in 33%, a Weber C fracture. Other indications were bimalleolar fractures of different types or including Volkmann fractures.

All patients in our study healed without any signs of chronic osseous infection like chronic fistula. Postoperative revision with implant removal after osseous consolidation of the fracture was performed in nearly all patients (97%). The mean time until removal of the implant was 8.10 ± 9.3 months (SD). Only in one patient the implant was left in situ.

The patients’ history was also reviewed for risk factors, such as smoking and comorbidities like diabetes and cardiovascular and thyroid diseases. The most common risk factors were cardiovascular disease (27%) and diabetes (17%). Generally, we could state 1.05 ± 0.2 risk factors per infection patient. In contrast, infection-free patients had statistically less risk factors at the time of surgical treatment (0.33 ± 0.1, *p* = .02) (Table 1).

**Table 1:** Epidemiological and treatment-related data of the patients (infection, matched-pair) included in the study

Criteria	Specification	Infection total		Follow-up		Matched-pair	
Total number of patients	Infection [*n*]	39		21		21	
Age	Total [year ± SD]	46 ± 17		43 ± 13		39 ± 16	
Gender	Male [*n*]	20	51%	13	62%	13	62%
	Female [*n*]	19	49%	8	38%	8	38%
Initial treatment	Primary [*n*]	21	54%	9	43%	21	100%
	Secondary [*n*]	18	46%	12	57%	0	0%
Diagnosis	Weber B [*n*]	15	38%	8	38%	8	38%
	Weber C [*n*]	13	33%	5	24%	5	24%
	Bimalleolar fracture, Weber B [*n*]	4	10%	3	14%	3	14%
	Bimalleolar fracture, Weber C [*n*]	3	8%	2	9.5%	2	9.5%
	Weber B and Volkmann [*n*]	3	8%	2	9.5%	2	9.5%
	Medial malleolus fracture [*n*]	1	3%	1	5%	1	5%
Microbiology	*Staphylococcus aureus* [*n*]	30	77%	19	90%		
	*Staphylococcus epidermidis* [*n*]	3	8%	1	5%		
	Beta-hemolyzing *Streptococcus* group A [*n*]	2	5%	1	5%		
	*Enterococcus faecalis* [*n*]	1	2.5%	0	0%		
	*Pseudomonas aeruginosa* [*n*]	2	5%	0	0%		
	*Providencia rettgeri* [*n*]	1	2.5%	0	0%		
Risk factors	Cardiovasc. disease [*n*]	11	28%	5	24%	2	10%
	Smoker [*n*]	6	15%	4	19%	1	5%
	Diabetes [*n*]	7	18%	4	19%	0	0%
	Thyroid disease [*n*]	5	13%	4	19%	1	5%
	Others [*n*]	12	31%	7	33%	3	10%
	No risk factors	20	51%	10	48%	15	71%
	Total per patient [*n* ± SD]	1.05 ± 0.2		1.1 ± 0.1		0.33 ± 0.1*	
Implant removal	Yes	38	97%	21	100%	16	76%
	No	1	3%	0	0%	5	24%

*p < .05

The data of 21 patients could be stated in our follow-up check-ups, including radiological and functional outcomes.

**Fig. 2:**
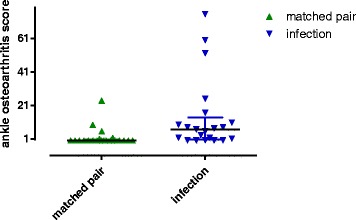
The matched-pair group showed significant better results in the Ankle Osteoarthritis Score compared to the infection patients

The radiological outcome, measured by the Ankle Osteoarthritis Scale, showed significant better results in the matched-pair group. Infection patients reached 13.9 ± 4.7 points, whereas the matched-pair group reached 2.0 ± 1.2 points (*p* = .02) (Fig. 2).

**Fig. 3:**
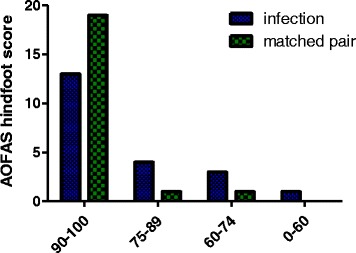
The matched-pair group showed significant better results in the AOFAS hindfoot score compared to infection patients

The AOFAS hindfoot score was used as a strict clinical rating system. The mean in patients with infection was measured with 87.3 ± 3.4 and in infection-free patients with 96.7 ± 1.9 (*p* = .02) (Fig. 3).

Patients who underwent surgical treatment because of implant-associated infections showed poorer outcomes in each scale of the SF-36. All sum scores were distinctly lower. In mean, patients without infection reached 91 ± 7.2 points in physical health and patients with infection 77 ± 22.8 points (*p* = .01) (Table 2).

**Table 2:** SF-36 showed significant reduction of several categories

Parameter SF-36	Infection	Matched-pair
Physical function*	85.2 ± 5.4	97.1 ± 1.2
Role physical	82.1 ± 7.9	97.6 ± 1.6
Body pain*	79.0 ± 6.4	97.2 ± 1.7
General health*	73.0 ± 4.8	87.5 ± 3.8
Vitality	67.4 ± 3.5	72.9 ± 3.2
Social functioning	88.1 ± 5.6	97.0 ± 2.4
Role emotional	90.5 ± 5.7	96.8 ± 2.2
Mental health	72.4 ± 2.3	75.8 ± 3.2
Physical health*	77.3 ± 5.0	90.9 ± 1.6
Psychic health	78.3 ± 3.8	86.0 ± 2.2
Total score SF 36*	79.7 ± 4.4	90.6 ± 1.5

*p* < .05

## Discussion

All patients in our study healed without any signs of chronic osseous infection like chronic fistula. Summarizing, the patients who suffered from an implant-related infection after a displaced ankle fracture had significantly poorer results than the matched-pair patients in all scores and outcome parameters. It was shown that they have significantly more risk factors. Moreover, we could show that the patients who underwent our standardized treatment were restored totally of the infection in all cases. We think that this is mainly attributable to the aggressive primary revision treatment. Although the infection patients showed significantly poorer results compared to the matched-pair patients, we could generally state a fair outcome in both, functional and radiological parameters in over 80% of the cases in patients after implant-associated infection of the ankle.

The aim of the operative treatment after ankle fractures is to restore a functioning joint that allows the patient to return to former level of activity. Some authors do not recommend operative treatment in elderly patients due to high complication rates, reported with up to 13% [26]. Compared to other complications, an implant-associated infection after ankle fracture and ORIF is rare but still one of the most feared complications [27]. The treatment is prolonged, expensive, and associated with an increased risk of the loss of function in the corresponding joint [28]. Other authors demonstrated that operative treatment in elderly patients showed significant better results in patient’s satisfaction [29]. We follow this opinion, treating most of the displaced ankle fractures operatively, except in patients with complex disorders or devastating pre-existing conditions.

According to our literature review, we did not find any data about the long-term outcome or an adequate consensus regarding the therapy of implant-associated infections of the ankle joint after fracture stabilization.

Most of the studies concerning infections of the ankle joint report on performed arthrodesis and prosthesis of the ankle joint or superficial wound infections [21, 30]. Other studies, reporting about plate osteosynthesis-related infections, mostly refer to long bones [31]. Therefore, we reviewed the outcome of our patients and brought our calculated therapy into question.

The overall success rate of our calculated treatment was excellent with osseous consolidation and infection-free wound conditions in all investigated patients. Previously reported success rates vary from 68 to 100% [9, 18, 19]. Zimmerli concludes that this variation is mainly contributed due to the type of antibiotics used [17]. All our patients got a firstly calculated and following specific antibiotic therapy during treatment after microbiological investigation. Aytac et al. consider the use of a permanent drainage in patients with osteomyelitis as an alternative treatment option, too. They showed promising results with a consolidation rate of 89%, investigating 67 patients with posttraumatic and postoperative osteomyelitis [20]. However, this calculated therapy with the use of a drain until osseous consolidation cannot be used for all patients. The prolonged use of the drain is rather a possibility if soft tissue conditions do not have to be treated with VAC devices or free flaps and if there is still the chance for osseous consolidation with the initial osteosynthesis after revision surgery and debridement of dead bone and soft tissue.

The need for a stable osteosynthesis is widely accepted for the treatment of implant-associated infections. Some authors disagree, recommending early removal of the implant for further reduction of bacterial load and biofilm [14]. In our experience, the need for a stable osteosynthesis is the main factor for following osseous consolidation. That is why the main principle in our treatment is to revise the patient with clinical infection signs as soon as possible and to reduce bacterial load and biofilm to suppress the infection until the fracture heals. Therefore, we use a drain until osseous consolidation to reduce bacterial load and operative revision with removal of necrotic tissue and lavage of the infected area if possible. However, patients should be aware of the daily care of their drain combined with strict postoperative care until osseous consolidation is reached.

Regarding the SF-36, we found consistent lower scores in the infection cohort than in the matched-pair cohort. Compared to a norm population, we could generally state a good outcome [22]. We also showed satisfactory results in functional and radiological scores. Therefore, we think that our calculated treatment is an efficient alternative method regarding long-term outcome. Nevertheless, compared to the matched-pair patients in our study, we had significantly lower scores in our infection cohort. This was stated in both functional and radiological. From these results, it must be concluded that patients after restored implant-associated infections have poorer long-term outcome, following arthritis and pain. This shows the importance of an aggressive initial operative approach and calculated therapy to get the patients to former activity level and also back to work after rehabilitation of the infection.

The number of risk factors has a significant effect on plate-related infections of the ankle after ORIF. Former studies showed that plate-related infections are also related to certain risk factors [12]. Diabetes and cardiovascular diseases are mentioned here as highly negative predictors, according to Sohoo et al. who described several risk factors in a 57,128 patient cohort [27]. Following Blooter et al., the risk of a deep infection after ORIF among diabetes patients is 16% [10]. Our results were consistent with these data, showing that diabetes and cardiovascular diseases are associated with a high risk for infections, too. Infection patients had significantly more risk factors per person than our matched-pair group in mean. Therefore, patients with one or more of the above-mentioned risk factors should be treated carefully after ankle fracture and ORIF or if the fracture morphology allows it with conservative treatment.

## Conclusion

Immediate revision surgery with radical debridement, microbiological diagnostics, and leaving a drain in place until osseous consolidation is reached with following removal of the implant in patients with implant-associated infections after ankle fracture and ORIF lead to cure of infection and fair long-term outcome in all cases. At the same time, a calculated antibiotic therapy must be given at least until soft tissue conditions have normalized. The success of this intervention could be stated by fair long-term results due to arthritis and functional outcomes as well as quality of life in patients with implant-associated infections after ankle fracture.

## Abbreviations

AO: Arbeitsgemeinschaft für Osteosynthesefragen
AOFAS: American Orthopaedic Foot and Ankle Society
ORIF: Open reduction internal fixation
SD: Standard deviation
SF-36: Short Form 36
